# Pore topology analysis in porous molecular systems

**DOI:** 10.1098/rsos.220813

**Published:** 2023-02-08

**Authors:** Verity Anipa, Andrew Tarzia, Kim E. Jelfs, Eugeny V. Alexandrov, Matthew A. Addicoat

**Affiliations:** ^1^ School of Science and Technology, Nottingham Trent University, Clifton Lane, Nottingham NG11 8NS, UK; ^2^ Department of Chemistry, Imperial College London, Molecular Sciences Research Hub, White City Campus, Wood Lane, London W12 0BZ, UK; ^3^ Samara Center for Theoretical Materials Science (SCTMS), Samara University, Ac. Pavlov Street 1, Samara 443011, Russia; ^4^ Samara Center for Theoretical Materials Science (SCTMS), Samara State Technical University, Molodogvardeyskaya Street 244, Samara 443100, Russia; ^5^ Laboratory of Coherent Optics, Samara Branch of P. N. Lebedev Physical Institute of the Russian Academy of Sciences, Novo-Sadovaya Street 221, Samara 443011, Russia; ^6^ Institute of Experimental Medicine and Biotechnology, Samara State Medical University, Chapayevskaya Street 89, Samara 443099, Russia

**Keywords:** topology analysis, porous organic cages, molecular packings

## Abstract

Porous molecular materials are constructed from molecules that assemble in the solid-state such that there are cavities or an interconnected pore network. It is challenging to control the assembly of these systems, as the interactions between the molecules are generally weak, and subtle changes in the molecular structure can lead to vastly different intermolecular interactions and subsequently different crystal packing arrangements. Similarly, the use of different solvents for crystallization, or the introduction of solvent vapour, can result in different polymorphs and pore networks being formed. It is difficult to uniquely describe the pore networks formed, and thus we analyse 1033 crystal structures of porous molecular systems to determine the underlying topology of their void spaces and potential guest diffusion networks. Material-agnostic topology definitions are applied. We use the underlying topological nets to examine whether it is possible to apply isoreticular design principles to porous molecular materials. Overall, our automatic analysis of a large dataset gives a general insight into the relationships between molecular topologies and the topological nets of their pore network. We show that while porous molecular systems tend to pack similarly to non-porous molecules, the topologies of their pore distributions resemble those of more prominent porous materials, such as metal–organic frameworks and covalent organic frameworks.

## 1. Introduction

Porous organic molecular materials are alternatives to extended porous materials, such as metal–organic frameworks (MOFs) and zeolites, for applications such as gas storage, molecular separations and catalysis because of their tunability and solution processability. Porous molecular materials form bulk materials in the solid-state, in either crystalline or amorphous forms, with structures governed by the interplay of weak intermolecular interactions between constituent molecular components. Unlike extended porous materials, the porosity of a molecular material results from ‘extrinsic’ porosity, which is defined by the voids created by the inefficient solid-state packing of the molecular components [1]. Alternatively, the molecules themselves can have an internal cavity, generating ‘intrinsic’ porosity in the solid-state; for example, with cucurbiturils [2] or cage-like molecules, with the latter having more than two entry or exit routes to the internal cavity [3]. With recent research effort, the porosity of molecular systems has even rivalled that of extended networks [4]. The solid-state packing of cage-like compounds determines, first, the presence of extrinsic porosity and, second, whether any intrinsic porosity is accessible. For example, intrinsic porosity will be accessible in the case where the cage windows align, but can be made inaccessible in the case where windows are blocked by the walls of neighbouring cages. The ‘efficiency’ of the solid-state packing, therefore, governs the overall porosity of the material. For a given molecule, it is difficult to predict its molecular packing and hence porosity prior to experimental testing. There are several examples of polymorphism influencing porosity [5], with reports of switching porosity ‘on’ and ‘off’ via solvent exchange [6]. It is also possible to control cage packing through solvent effects [7], modification of external chemistry [8] and co-crystallization [9,10]. Through crystal structure prediction methods, it has become possible to computationally predict the most likely crystal packings for a given porous molecule [10–12].

Crystalline, extended porous materials are often defined by their underlying topology, which describes the connectivity of the components of the network. The topological analysis of frameworks allows for a simplified categorization [13], and analysis of their structures [14] and porosities [15–20]. In this work, we apply the Reticular Chemistry Structure Resource (RCSR) [21] definition of topology [22], which has become well-known in the field of MOFs through the idea of isoreticular structure design based on the underlying topological net of the framework [23]. Similar analyses have been applied to non-covalently connected species [24] to yield insight such as the distribution of regular hydrogen bonds [25], the packing of small molecules compared to proteins [26] and to determine the relative importance of different intermolecular interactions in sulfonamides [27]. One recent study generated energy–structure–function (ESF) maps for porous molecular crystals [28]. However, topological analysis of the porosity and packing of porous molecular materials has not yet been performed. Yet, the topology of the migration pathways within porous molecular materials is fundamental to their utility as porous materials. For example, polymorphism would be evidenced by a change in the topology of migration pathways; breathing can be shown by a change in the migrating probe of extrinsic migration pathways; and responsive behaviour of molecules would be illustrated by a change in the migrating probe of intrinsic channels.

The molecular-level topological landscape of porous organic cages (POCs) has previously been detailed, where topology was defined as ‘the underlying connectivity of molecular building blocks in the molecular cage, which is unchanged upon any physical deformation’ [29]. This definition only describes the connectivity of a single cage and gives no information regarding the spatial relationship of cages with respect to each other. Unlike extended materials, it is possible to define multiple ‘levels’ of topologies in porous molecular materials in the solid-state, which do not, by definition, have a concrete relationship between them. The archetypal POC, **CC3** [8], formed by a [4 + 6] cycloimination reaction of four 1,3,5-triformylbenzene units with six (*R*, *R*)-1,2-diaminocyclohexane linkers yields a diamondoid (**dia**) network of connected pores in the solid-state [30]. In this example, the four windows defined by the tetrahedral molecular topology align during crystal packing to form a porous network. However, **CC1**, with the same molecular topology as **CC3**, can form a non-porous structure in the solid-state, where the cage windows do not align to connect the isolated pores [6].

In this work, we introduce an analysis of the pore topologies of porous molecular materials in the solid-state using ToposPro [31], which we apply to a large dataset of X-ray diffraction crystal structures of porous molecular materials. The complex correlations between molecular porosity and material porosity have recently been explored using geometrical descriptors [32]. Through our analysis, we show relationships between the different levels of distinct topologies present in porous molecular materials, with a goal to develop guiding principles for the formation of materials with target properties. Furthermore, we attempt to use the underlying topological nets to determine if it is possible to apply isoreticular design to porous molecular materials. Overall, our automated analysis of a large dataset gives a general insight into the relationships between the different topological nets [22,33,34]. Our analysis also allows comparison of the pore topologies of porous molecular materials to those of extended porous materials, such as MOFs and zeolites [35,36].

## 2. Methods

### 2.1. Database generation

We have assembled a database (available at https://github.com/andrewtarzia/cage_collect/) of organic crystal structures containing at least one molecule with an intrinsic void. For each porous molecule, the centre-of-mass (COM), centre-of-pore (COP) and centre-of-window (COW) positions were all identified and marked using pyWindow. The COP position is refined from the COM and differs typically only in non-symmetric cages (i.e. where a functionalized linker distorts the COM from the geometric COP) [37]. Full details of the database generation can be found in electronic supplementary material, §S1. This cleaned database was then analysed using ToposPro [31].

### 2.2. Topological analysis

To undertake a topological analysis of the crystal structures, we used several different approaches (each described below) to construct topological nets that show distinct aspects of the connectivity motifs in the crystal structures. In this way, we can explore the connections between the distinct aspects that can govern the packing of cage-like molecules.

#### 2.2.1. General workflow description

**Figure 1.**
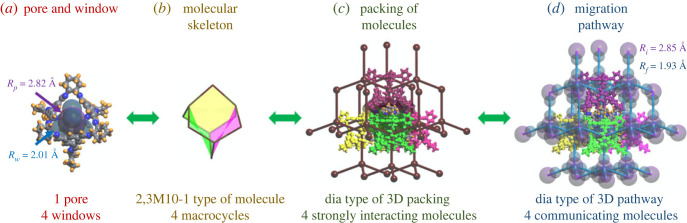
The geometrical and topological analysis of porous molecular systems from local to overall in four general interrelated steps: (*a*) the chemical bonds, pores and windows within a molecule are identified, (*b*) a molecule can be known by the similarity of its topology and shape with other compounds, (*c*) molecular packing in the porous molecular system is assigned to known topological types, and (*d*) migration pathway topology can be classified into different groups according to its branching and ability to exchange guest molecules directly.

The geometrical and topological analysis of porous molecular systems was arranged in the logic ‘from local to overall’ and can be summarized by four general interrelated steps. The details for computing physical parameters of the systems are given in §2.2.2–2.2.7 and in this section, we describe the overall physical picture of the analysis and illustrate it by flowchart in figure 1 with the example of the well-known porous molecular system **CC3** (Covalent Cage 3), Refcode FOXLAG [8].

First, chemical bonds, pores and windows within a molecule should be identified to make sure that the structure under consideration belongs to the class of porous molecular systems. The structure of **CC3** (FOXLAG) consists of four 1,3,5-trimethylidenebenzene groups enclosed in four cycles with six (*R*, *R*)-1,2-diaminocyclohexane spacers, and it has four windows and one pore with radii of *R*_*w*_ = 2.01 Å and *R*_*p*_ = 2.82 Å, respectively.

Second, using information about chemical bonding of building blocks, porous molecules can be grouped by the similarity of their topologies and shapes. The structure of **CC3** molecule has adamantane-like topology, with 1,3,5-trimethylidenebenzene groups in the 3-connected corners and (*R*, *R*)-1,2-diaminocyclohexane spacers at the 2-connected edges, and this topology predetermines the existence of four macrocycles.

Third, molecular packing, defined from the information about valence bonds within each molecule and weak interactions with surrounding molecules, in the porous molecular systems can be assigned to known topological types. The molecular packing of **CC3** can be described as a diamondoid-like structure since: each of the four macrocycles is contacting with one other molecule by the largest area of interactions; the molecular coordination number equals the number of macrocycles; and their orientation follows tetrahedral geometry that is typical for diamondoid-like structures.

Fourth, the migration pathway topology, crucial for practical applications, can be classified into different groups of branching based on the information about availability of the windows and pores, as well as intermolecular interactions, since only contacting molecules can exchange guest molecules directly, without losing the guest to external pores. The diamondoid topology of the strongest intermolecular interactions, the close proximity of macrocycles and their large-enough size enable **CC3** to make the most of the opportunity of porous space inside molecules; that topology follows maximally possible branching of 4-c diamondoid motif, and the widest cavity and channel of *R*_*w*_ = 2.85 Å and *R*_*f*_ = 1.93 Å, respectively.

Using this logic, it can be seen that when combined, the four steps of geometrical and topological analysis comprise a complex picture of structural interrelations governing the organization and functionality of the porous crystalline solid, starting from finite objects and finishing with periodic nets that are important for developing strategies of designing new porous molecular systems. We consider this approach in detail in the following sections.

#### 2.2.2. Molecular skeleton topology

We considered the topological type of the molecular topology (named the ‘skeleton topology’ from here on). It is the simple representation of the cage in terms of points of extension and linkers (spacers), which are classified using the NDk nomenclature of ToposPro [31]. This nomenclature gives the same information as both the ‘*N*-(connected) building block and *N*, *M*-net’ nomenclature often used to describe MOFs, and the $X_{p}^{m}Y^{n}$ nomenclature of POCs [29]. A detailed description of the NDk nomenclature is included in electronic supplementary material, §S2. For example, the cages **CC1**–**13** all belong to the same topological type, 2,3M10-1, which is a type of adamantane-like cage observed in 150 of our 1033 structures, and they are composed of four 3-c vertices, joined by six 2-c (ditopic) linkers yielding a **Tri**^4^**Di**^6^ topology (electronic supplementary material, figure S1).

To determine the skeleton topology from the molecular coordinates only, atoms are clustered into building blocks using the same method as used to deconstruct MOFs into building blocks and implemented in ToposPro [35]. A more detailed description is included in electronic supplementary material, §S3.

#### 2.2.3. Molecular packing topology

**Figure 2.**
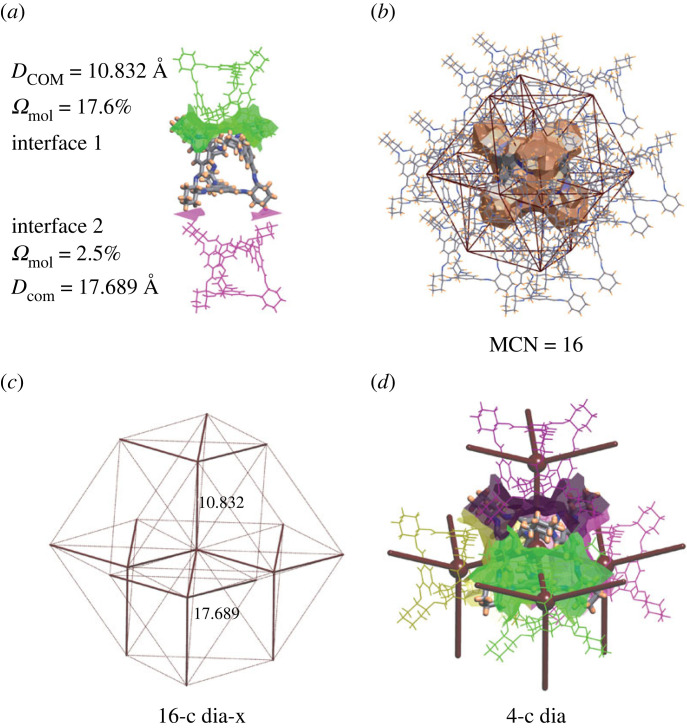
Building the underlying net of molecular packing and net of strongest intermolecular interactions for the structure with Refcode FOXLAG. (*a*) Interfaces of two types constructed from atomic Voronoi faces with solid angles $\Omega_{\mathrm{mol}}=17.6\text{\%}$ (green) and 2.5% (pink), as well as distances between the COMs of the molecules *D*_COM_ = 10.832 Å and 17.689 Å. (*b*) Molecular Voronoi polyhedron and 16-molecule environment surrounding the central molecule, and corresponding underlying net of COMs and intermolecular interactions, yielding a molecular coordination number (MCN) of 16. (*c*) 16-c underlying net of topological type **dia-x** with thin and thick lines representing edges between COMs of weakly ($\Omega_{\mathrm{mol}}=2.5\text{\%}$) and strongly ($\Omega_{\mathrm{mol}}=17.6\text{\%}$) interacting molecules, respectively. (*d*) Strongly interacting four molecules and intermolecular interfaces highlighted in different colours surrounding a central molecule and corresponding 4-c underlying net of **dia** topology (brown lines).

The underlying net of molecular packing was established by ToposPro [31] as described in [26]. For this, valence and non-valence interatomic interactions were identified with the ‘Domains’ method [38]. The molecules were identified as parts of a structure connected by valence bonds, while the molecular environment and strength of intermolecular interactions were determined from construction of the molecular Voronoi polyhedrons [24,39]. Two molecular Voronoi polyhedrons interact when they have a common external face, constructed by faces of atomic Voronoi polyhedrons on the interface of two molecules (figure 2*a*). Counting the molecules forming the interface around a central molecule gives the molecular coordination number.

The underlying net of molecular packing (figure 2*b*) is constructed by representing each molecule by a node at the COM and drawing a single simple edge between two COMs. Such an underlying net focuses on the connections between building units, which helps comparisons of structural topological motifs for different compounds and allows for grouping them into isoreticular series defined by their topological types [22,35]. To denote nets, the same nomenclature as commonly used for MOFs is employed, *viz.* most nets are described by the RCSR three-letter [21] and the TTD collection NDk [35] symbols, but Fischer’s symbols of 3D (e.g. 6/4/c1), 2D (e.g. *K*I*a*) and 1D (e.g. (4,4)(0,2)) sphere packings [40,41], EPINET nets (e.g. *sqc36*) [42], or Blatov’s subnets (e.g. **fcu**/cubic closest packing; *Fm* − 3*m* → *C*2/*c* (*b* − 2*c*, *a*, − *b*; 1/4, 0, 1/4); Bond sets: 1,2,3,4,5,7,8,9: **fcu**) are also used [43].

#### 2.2.4. Molecular packing topology of strongest interaction

The edges of the underlying net may correspond to intermolecular contacts of different geometries and strengths. Ignoring the weakest interactions will reduce the connectivity of the underlying net. The accumulative value of intermolecular interactions and the geometric arrangement of molecules in a local environment can be measured using one parameter, termed the solid angle of the intermolecular interface ($\Omega_{\mathrm{mol}}$). $\Omega_{\mathrm{mol}}$ corresponds to the intermolecular contact area, which has been shown, in the absence of particularly strong intermolecular interactions (e.g. hydrogen bonds), to correlate well with the interaction energy [26,44–48]. Similarly, we reduced the connectivity of the underlying net to the level of strongest intermolecular interactions, so edges are related to the extrinsic faces of the largest $\Omega_{\mathrm{mol}}$ within a periodic structure. For example, the structure with Refcode FOXLAG has intermolecular interactions of two types: the strongest with solid angle $\Omega_{\mathrm{mol}}$ of 17.6% and the weakest with $\Omega_{\mathrm{mol}}$ of 2.5% (figure 2*a*). Removing edges of the underlying net related to both types of interactions disjoins the net into separate nodes. Removing the edges related only to the weak interactions reduces the connectivity of the net from 16 to 4 (figure 2*c*,*d*), and the net keeps the periodicity as 3D. For FOXLAG, the periodicity cannot be further reduced without losing the periodic structure. In this case, we chose the 4-c net as the net of strongest interactions. In other structures, a net of strongest interactions can have lower dimensionality, i.e. 2D or 1D. Such a representation helps to reveal the underlying net of the most important interactions. For example, we can find all structures and porous molecules where interactions between faces are the strongest, and they predetermine the formation of diffusion pathways through cages.

#### 2.2.5. Migration pathway topology

**Figure 3.**
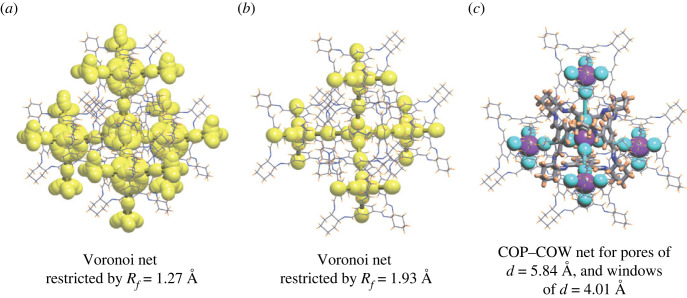
Three different representations of the pore connectivity for the structure with Refcode FOXLAG. (*a*) Yellow balls and edges show a migration pathway elucidated from the Voronoi net by removing all vertices and edges closer than a channel width *R*_*f*_ = 1.2722 Å to the van der Waals radius of neighbouring atoms. (*b*) The 3D Voronoi subnet located farther than *R*_*f*_ = 1.9328 Å to the van der Waals surface. (*c*) The net of connections between the pore centre (COP atom; purple ball) and the centre of the windows (COW atoms; cyan balls), COP–COW and COW–COW edges. Graphs of molecules are shown by thin sticks; in (*c*) the central molecule is highlighted by bold sticks.

The search for migration pathways in a crystal structure was performed by constructing its Voronoi net [49–51]. Each vertex of a Voronoi net is located in the centre of an elementary void and each edge is the elementary pathway between two voids. We scanned the Voronoi net for the vertices and edges distanced more than 1.2 Å from the van der Waals surface of atoms in the structure. This representation finds the whole map of potentially solvent-accessible channels and voids in the structure (figure 3*a*).

#### 2.2.6. Migration pathway topology of largest probe size

By further increasing the size of the migrating probe radius, the map may be reduced to 3D, 2D or 1D (figure 3*b*) [51], and accordingly yields the largest possible size of a migrating probe, which can travel in three, two or only one dimension, respectively.

#### 2.2.7. Pore-to-window pathway (COP–COW) net

The above four approaches have been previously described and employed elsewhere. However, we also used a new type of net, termed the ‘COP–COW’ net, consisting of connections between dummy nodes positioned at the COP and COW as calculated by pyWindow [37] (figure 3*c*). To define this net, the following criteria were used:

1. The COP node of a molecular pore has edges only with COW nodes of windows of the same molecule.
2. COW window nodes of one molecule can have only one edge with a COW node of another molecule; this will be the shortest one.
3. The COW–COW edge does not intersect with the van der Waals spheres of any atom of the structure.
4. The COW–COW edge is added only if a direct migration pathway exists between pores of the two cages. Direct pathways mean that edges and nodes of the Voronoi net connecting pores of the two molecules are formed by only Voronoi polyhedrons of the two molecules, and the edges and nodes do not have connections to Voronoi edges and nodes of any other molecule. In other words, there is no gap between molecules where the migrating probe can go outside of the elementary channel between two molecules.

**Figure 4.**
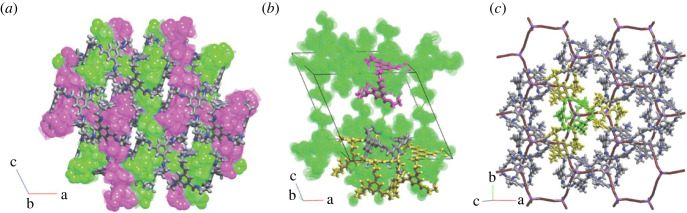
Crystal structure with Refcode OFOQAE and its representations of migration pathways: (*a*) the two interpenetrating 3D pore networks derived from the Voronoi net (shown by magenta and green balls); (*b*) one of the 3D pore networks (in green) following which motif a hydrogen guest molecule would freely migrate in the crystal space; and (*c*) in purple, the 2D 3-c COP–COW net of **hcb** topology, where a hydrogen guest molecule would go through only the cages without passing through extrinsic voids. The central and communicating cages are highlighted in green and yellow, respectively.

For example, the structure with Refcode OFOQAE has a 3D 4-c **dia** topology from the COP–COW net, but after cutting one COW–COW edge that does not satisfy rule 4 above, the topology becomes 2D 3-c **hcb** (figure 4), which means that the topology of the channels going through only the molecules without coming out to the space outside of the molecules is actually 2D **hcb**. The molecules from neighbouring **hcb** layers are more distanced from each other and have gaps, while molecules within the **hcb** layer are close to each other and have continuous pores between windows. This COP–COW net represents then, in the simplest way (in comparison to Voronoi nets), potential connectivity of pores and windows of the molecules and can also be classified into topological types in accordance with conventional nomenclature.

Altogether, the hierarchical description of molecules and their solid-state packing enable one to reveal the geometrical–topological relations that drive the assembly of molecules with a given number and spatial arrangement of windows into molecular packings with specific topologies and migration pathways. The information about topological classification of the skeleton topology, underlying nets, Voronoi nets and COP–COW net is presented in electronic supplementary material, spreadsheet.

## 3. Results and discussion

### 3.1. Skeleton topology of the individual porous molecules

**Figure 5.**
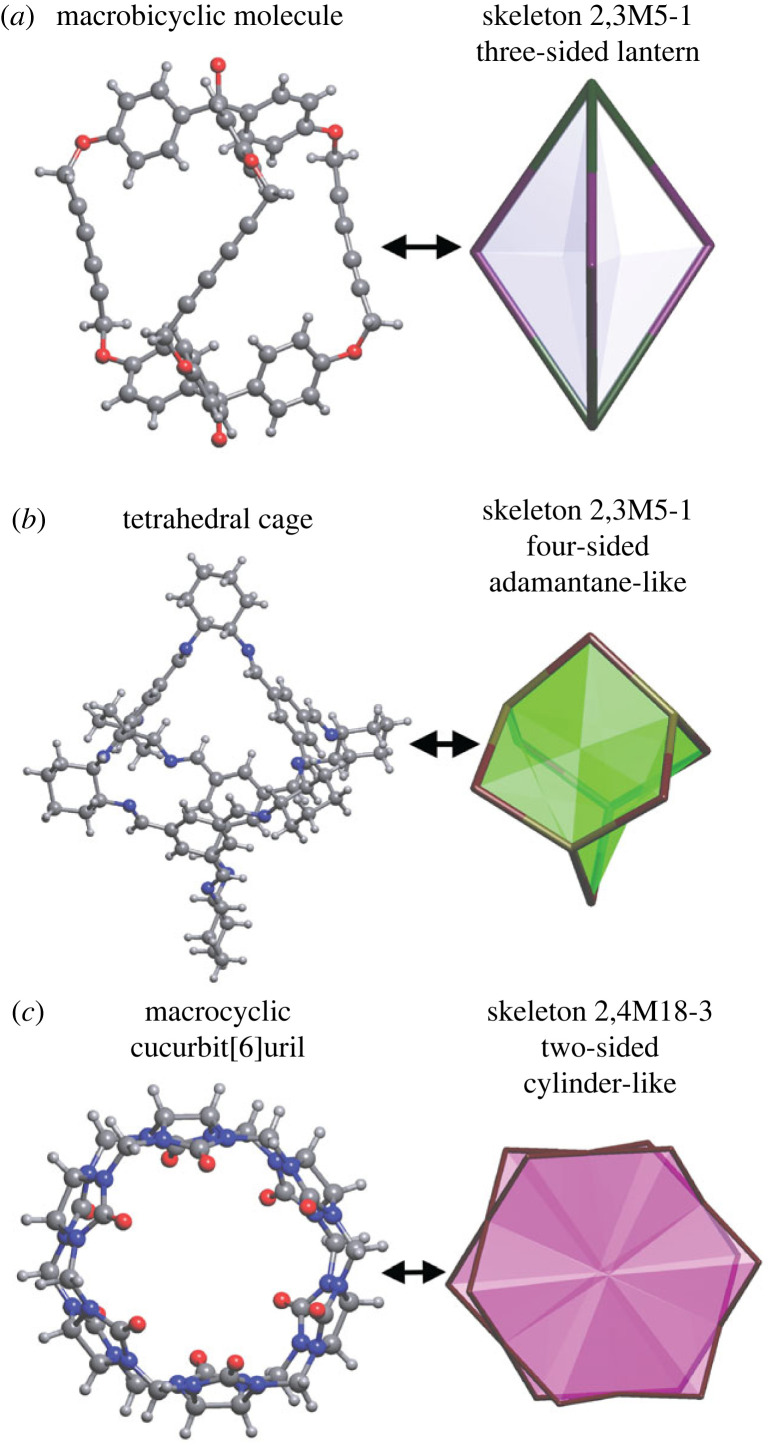
Frequently occurring molecular cage skeletons, shown with an example molecule: (*a*) a macrobicyclic molecule from the structure REFVON (hydrogen atoms of hydroxyl groups were not determined) [52]; (*b*) a tetrahedral cage (**CC3**) from the structure FOXLAG [8]; and (*c*) a macrocyclic cucurbit[6]uril from the structure JIJNEY [53].

We begin our analysis by describing the intrinsic features of the molecules present in our dataset. An understanding of these features is important to know the molecule types that our analysis is applicable to. The individual molecules in the crystal structures under consideration in this work can be grouped according to their topological and geometrical parameters, such as skeleton type, number of macrocycles in the skeleton, number and size of windows, and the size of their intrinsic pore. The skeleton topology, or molecular topology [29], of a porous molecule predetermines the number of possible windows in the molecule and its underlying shape. In our database, we found 42 different types of skeleton topologies in 720 distinct molecules, with the number of atoms ranging from 35 to 972. The most frequent skeleton types are 2,3M5-1 (count of 245), 2,3M10-1 (154) and 2,4M18-3 (115), which are depicted in figure 5. For example, the diyne-bridged macrobicyclic molecule shown in figure 5*a* is of type 2,3M5-1 and is reminiscent of a three-sided lantern [52]. Imine-linked cages come in a variety of molecular skeletons [29], but the archetypal tetrahedral cage (**CC3**) synthesized via the condensation of 1,3,5-triformylbenzene with (*R*, *R*)-1,2-diaminocyclohexane shows the adamantane-like skeleton 2,3M10-1 with four windows (figure 5*b*) [8]. Our dataset includes many tubular or macrocyclic molecules with intrinsic porosity, such as cucurbiturils, pillarenes and cyclodextrins. Figure 5*c* shows the skeleton topology (2,4M18-3) of cucurbit[6]uril with two windows on opposite sides of the cylinder-like molecule [53].

Through the modification of the size, shape and/or chemistry of the precursors, it is possible to tune the intrinsic porosity of a molecule. Often, this corresponds to tuning the pore and window sizes, which are the two geometrical properties of a molecule that are closely linked to the performance of the solid-state material [54]. By design, our sample of porous organic molecules consists of molecules formed from relatively rigid, aromatic building blocks connected by aliphatic, ether or amine linkers, which includes molecule subclasses such as POCs, pillarenes, cyclodextrins and cucurbiturils. The observed numbers of windows in the structures are 1–8 and 12, of which 2, 3 and 4 windows are most frequent. The pores and windows have diameters in the ranges 0.2–16.0 Å and 0.3–20.5 Å, respectively. The majority (78%) of the structures have pore and window diameters larger than 2.4 Å, which makes them accessible to hydrogen (kinetic diameter: 2.4 Å) [55]. Approximately 26% and 15% of the windows and pores, respectively, of molecules in our dataset are not accessible to hydrogen when the static structure alone is considered. Molecules with the same skeleton topology were found to have both wide (greater than or equal to 2.4 Å) and narrow (less than or equal to 2.4 Å) window sizes, showing that the pore and window size is not an inherent feature of a particular skeleton topology and that both pore and window size can be tuned while maintaining a specific skeleton topology. It should be noted that the number of windows is not always the same as the number of macrocycles in a molecule because some macrocycles are collapsed and cannot be penetrated without distortion. Alternatively, some macrocycles can give two or more windows due to an irregular twisting of the macrocycle or some other part of the molecule pointing into the macrocycle (see electronic supplementary material, figure S3). In the above cases, a porous molecule is often deemed to not be shape persistent, which suggests that the material will be non-porous after desolvation.

### 3.2. Describing the local environment of porous molecules

Both the shape and functional groups present in a molecule are important factors in determining the intermolecular interactions that are possible for that molecule and thus its local environment during crystallization [56,57]. We quantify the local environment using the molecular coordination number, determined by the solid angle of the intermolecular interface ($\Omega_{\mathrm{mol}}$), as previously described. The simplest topological property associated with a molecule’s local arrangement is its molecular coordination number, which is distributed over a wide range (2–27) for the molecules in our dataset, with coordination numbers of 12, 14 and 16 occurring most frequently. Data for all molecules is included in the electronic supplementary material, spreadsheet.

**Figure 6.**
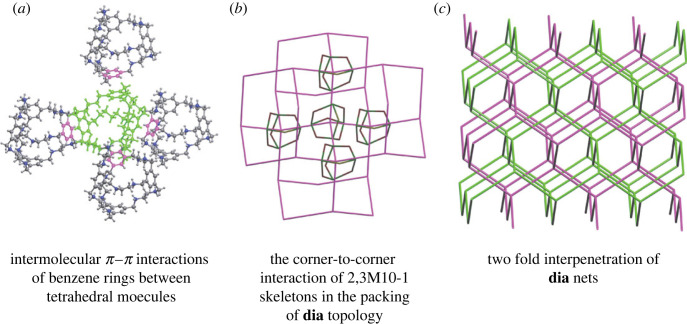
Molecular packing of tetrahedral cages with a solid angle ($\Omega_{\mathrm{mol}}$) between molecules of more than 15.0% in the structure with Refcode OVENEK [58]. (*a*) Central molecule is shown in green and neighbouring molecules in grey, with *π*-stacking phenyl groups shown in pink. (*b*) Cage skeleton shown in grey and **dia** net shown in purple. (*c*) Two interpenetrating **dia** nets shown in green/pink.

For example, the structure with Refcode FOXLAG and skeleton topology 2,3M10-1, as previously described, has 16 molecules in its local environment, but these exist in two distinct sets of 12 and 4 equivalent neighbours. This motif was observed for several cages, which are listed in electronic supplementary material, §S5. Another example, in the structure with Refcode OVENEK [58], *π* · · · *π* intermolecular interactions between benzene rings orient the positively charged tetrahedral molecules to have corner-to-corner interactions, while the cavities between the cages are filled with sulfate anions (figure 6*a*,*b*). Similar corner-to-corner orientations were found in three other structures with molecular skeletons of the 2,3M10-1 type (FIFTEV, OVENIO and OVENOU).

The relative orientation of the molecular windows in the local environment determines whether the intrinsic pores of the interacting molecules are accessible to each other. An intrinsic migration path, by definition, requires face-to-face contacts. Corner-to-corner, edge-to-edge, corner-to-edge, corner-to-face and edge-to-face contacts all require that a diffusing species transits through extrinsic pore space to reach a neighbouring cage molecule. For example, in the structures PIFTOQ and PIFVUY with macrobicyclic molecules of type 2,3M5-1 (skeleton topology shown in figure 5), the cages have windows and intrinsic pores large enough for penetration by a hydrogen molecule [59]. However, all three windows are blocked by edges of the three closest neighbouring cages. We analyse other window-blocked molecules in electronic supplementary material, §S9.

### 3.3. Describing the periodic packing of cages

Extending beyond the structure of the local environment surrounding a single molecule, we can describe the topology of the periodic packing of porous molecules in crystal structures. For example, the two structures discussed above, FOXLAG (figure 2) and OVENEK (figure 6), show similar supramolecular clusters of one central cage molecule with four strongly interacting surrounding molecules that are arranged tetrahedrally, which produces the 4-c **dia** topology (based on the connectivity of only the strongest intermolecular interactions). We note that the OVENEK structure is less dense and has two interpenetrating **dia** nets (figure 6*c*). A different representation of molecular packing can be achieved by using all (strong and weak) interactions to define the connectivity between cages. In the above examples, the topologies ascribed to the molecular packing based on all interactions are different than those with just the strongest interactions; FOXLAG and OVENEK have 16- and 14-molecule local environments, which produce 16-c **dia-x** and 14-c **bcu-x** topological motifs, respectively.

**Figure 7.**
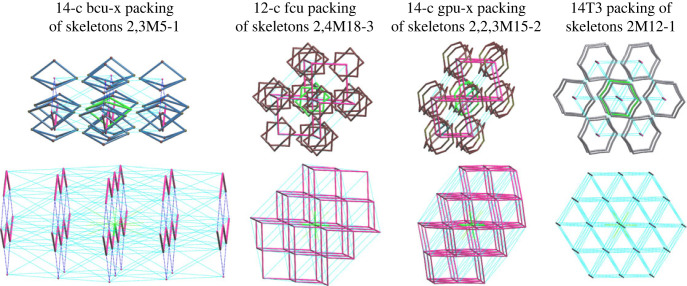
Top: examples of the most frequently occurring pore topologies in the crystal structures between skeletons 2,3M5-1 (Refcode EQEXOP), 2,4M18-3 (Refcode MUNBAA), 2,2,3M15-2 (Refcode QACPOB) and 2M12-1 (Refcode NUQVAY), and formed by four different 12-coordinated and 14-coordinated molecular environments. The skeletons are placed with the centres in the nodes of the underlying nets **bcu-x** (body-centre uninodal), **fcu** (face-centre uninodal), **gpu-x** (*γ*-polonium uninodal) and 14T3, respectively. The central skeleton is highlighted in green. Bottom: the extended fragments of the underlying nets **bcu-x**, **fcu**, **gpu-x** and 14T3. Thick lines highlight the edges of the nets of interactions with $\Omega_{\mathrm{mol}}\geq 7.5\text{\%}$: 2C1, **pcu**, **hex** and 2C1, respectively. The node in green is the central node.

In general, porous molecules have the same frequently observed topologies as seen in the packing of organic single molecule or co-crystal structures [24]. From 319 types of observed nets derived from all interactions in a crystal structure, we found that the most common four topologies are **bcu-x**, **fcu**, **gpu-x** and 14T3 (figure 7 and electronic supplementary material, S7) [26]. These nets have high coordination numbers of nodes, ranging from 12 to 14. This distribution reflects the general trend in molecules crystallizing in the most symmetrical arrangement possible for the given shape and intermolecular interactions [24]. Artificially removing solvent molecules from the crystal structures in our dataset affects only some particular cases by leaving an unphysical amount of empty space and reducing the network topology from 3D to 2D (three structures) or 1D (one structure), but the overall statistics do not suffer from this artificial structure reduction. Thus, the arrangement of molecules in structures with interstitial solvent molecules in general is similar to structures crystallized without solvent molecules at all [26].

Interestingly, the distribution of nets derived from only the strongest interactions observed in each structure (i.e. the highest $\Omega_{\mathrm{mol}}$) is much narrower, with only 62 distinct topology types found. In this case, nets with minimal coordination numbers (2–4) are the most frequent: 2C1 (simple chain), **sql** (square lattice), **hcb** (honeycomb lattice), (4,4)(0,2) (ladder) and **dia** (diamondoid net).

#### 3.3.1. Polymorphism

The packing of molecules depends on their structure and composition, as well as the crystallization conditions such as the solvent choice. To consider how we could use topological indicators to show polymorphism, we examined our database and found 67 molecules that form a total of 191 different molecular packings (we did not take into account any clathrate and counterion species present). For example, the adamantane-like cage **CC3** occurs in eight different crystal structures, including co-crystals with other porous molecules or larger solvent molecules, which results in structures with different molecular packings (Refcodes: DEDTUE, DEDVEQ, HEVQOQ, HEVQUW, HEVRAD, MOTZOM, MOVBUW and NOLSEO). The net of strong interactions ($\Omega_{\mathrm{mol}}\approx 7.5\text{\%}$) was found to have topologies **dia**, **hcb** or **unc** in the *p*-xylene, dichloromethane-methanol-hydrate and mesitylene clathrates of HEVRAD, MOTZOM and HEVQOQ, respectively.

A packing assembly with a combination of face-to-face and open-window orientations of cages restricts the periodicity of the intrinsic migration pathways. Thus, the structure of HEVRAD does not have any extrinsic pathways since the cages are densely packed with a face-to-face orientation of all the windows producing 3D intrinsic migration pathways. In contrast, the open windows in the structures of MOTZOM and HEVQOQ mean that the intrinsic pores communicate with extrinsic pathways through one and all four windows, respectively. As a result, in the two latter structures, the intrinsic migration pathway has 2D periodicity (MOTZOM) or does not exist at all (HEVQOQ).

### 3.4. Describing migration pathways

Here we describe the migration pathways through a porous structure using two distinct nets, the COP–COW net and the Voronoi net, which represent distinct aspects of the accessibility of the porosity in a crystal structure. The COP–COW net describes the topology of the connection of the intrinsic pores of the molecules in a structure through face-to-face (window-to-window) contacts. From the distribution of the COP–COW topologies, we can see that more than 84% of structures do not have a periodic system of intrinsic channels, while 1D periodicity (9%) is much more frequent than 3D (5%) and 2D (1%) periodicities. Unique cases are 3D interpenetrating (e.g. FIFTEV) and mixed 2D + 1D systems (e.g. PUDXES04). From the 15 observed topological types, the most common are chain-like topologies 2C1, framework-like topologies **dia** and **bor**, and 2D topologies **hcb**. As we will see below, these topologies are the result of the design of cages and tuning of their packing.

The Voronoi net of the accessible channels shows the periodicity of the porous space that is accessible to a H_2_ molecule (with minimal probe radius 1.2 Å) and provides information about the sizes of the widest channels and cavities. From the distributions of the radii of the widest cavity (*R*_*i*_), channel (*R*_*f*_), window (*R*_*w*_) and intrinsic pore (*R*_*p*_) over all structures (electronic supplementary material, figure S2), we can see intrinsic pores and windows follow the trends of widest cavities and channels, with maxima at about 2.4 Å (*R*_*i*_), 2.4 Å (*R*_*p*_), 1.6 Å (*R*_*f*_) and 1.8 Å (*R*_*w*_). However, in 72% of structures *R*_*w*_ > *R*_*f*_ and in 59% of structures *R*_*p*_ < *R*_*i*_, which means that the accessibility of windows is usually restricted by the molecular environment due to the packing of molecules, and very often the extrinsic pores are larger than the intrinsic pores (electronic supplementary material, figure S2). It should be noted that 28% of structures do not have any periodic channels that are accessible to a hydrogen molecule when the static structure is considered. In contrast to the intrinsic migration pathways defined by the COP–COW net, 3D migration pathways (40%) are the most common Voronoi nets, while 1D (18%) and 2D (14%) periodicities are much less common. This apparent discrepancy leads us to consider the balance of intrinsic and extrinsic porosity in more detail, with the goal to understand the possible routes to control these two factors.

**Figure 8.**
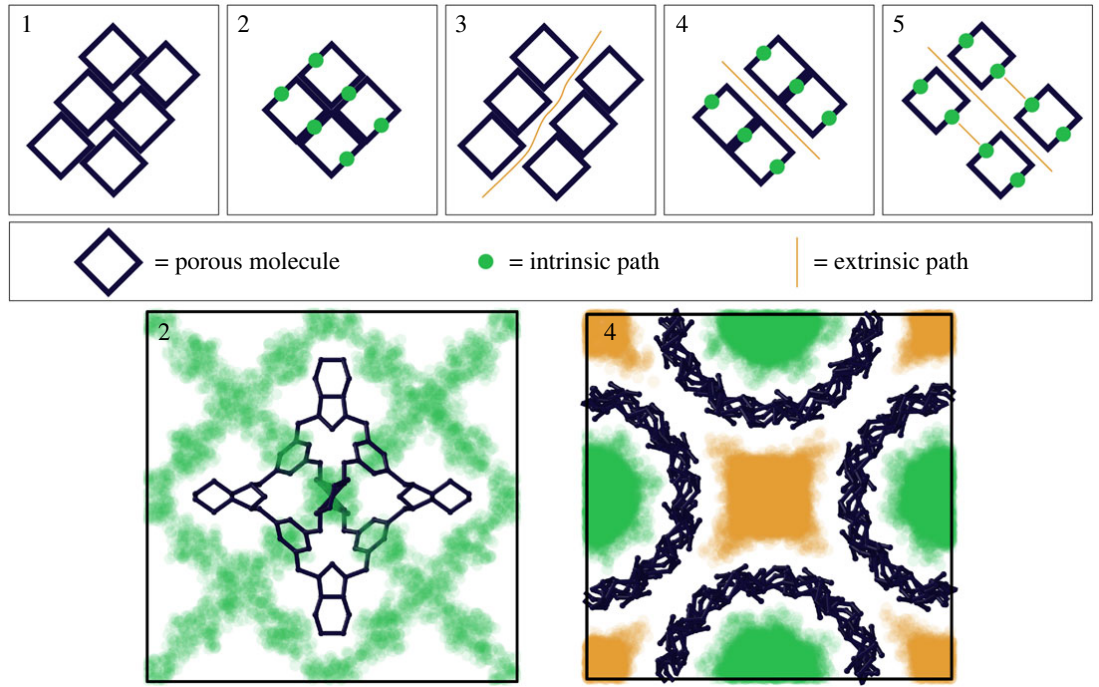
Schematics of the five classifications of pore networks described in the text: (1) no migration pathways; migration pathways are restricted to (2) intrinsic or (3) extrinsic pores, respectively; (4) there are two distinct migration pathways that coexist; and (5) there is one migration pathway made up of intrinsic and extrinsic pores. Paths of intrinsic and extrinsic porosity are coloured green and yellow, respectively. Examples from the porous molecule database that represent categories 2 (CAXFIT) and 4 (NUNRIX) show the distinction between the intrinsic and extrinsic porosity in these classifications. Note that for clarity, only one cage is shown in the example of category 2, where the windows of surrounding cages are arranged face-to-face with the windows of the shown cage producing an intrinsic pore network. Visualization of the pore network was calculated using Zeo++ [50].

Together, the COP–COW and Voronoi nets help to define the categories of accessibility of the intrinsic and extrinsic pores in the crystal structures (figure 8). We can propose the following five categories of porous structures:

1. There are no migration pathways because the molecular packing has no accessible pores.
2. Migration pathways are constructed only by the intrinsic pores and windows of porous molecules.
3. Migration pathways are constructed only by the extrinsic space resulting from the molecular packing.
4. There are two distinct migration pathways, made up of intrinsic and/or extrinsic pathways, which coexist and do not communicate.
5. There is one migration pathway made up of intrinsic and extrinsic pores.

The 1033 structures in our dataset are sorted into the five categories listed above using the COP–COW and Voronoi nets, restricted by *R*_*f*_ ≥ 1.2 Å and the following criteria:

1. Category 1, made up of 291 structures, corresponds to the case where there is no Voronoi net or the Voronoi net is 0D. The absence of the Voronoi net or its 0D periodicity means that a structure does not have any periodic system of accessible channels. In the case of porous molecules, this can indicate that their windows are blocked by neighbouring molecules. Importantly, this does not rule out the presence of intrinsic or extrinsic, non-periodic porosity, where the pathway does not traverse the entire unit cell of the crystal structure. For example, in the structure GANDOQ, with closely packed adamantane-like cages, only a migration pathway between a pair of two molecules that share a single face was found (electronic supplementary material, figure S5).
2. Category 2, made up of 90 structures, corresponds to the case where the periodicity of the Voronoi net is 1D, 2D or 3D and coincides with the periodicity of the COP–COW net. Further, the Voronoi net passes through the intrinsic pores of the molecules in the structure, and the edges and nodes of the Voronoi net are formed by atoms on the interior of one molecule or by pairs of molecules connected by COW–COW edges (where COW vertices define the windows of the molecules). The examples of structures FOXLAG (figure 3) and HEVRAD in this category are considered above.
3. Category 3, made up of 199 structures, corresponds to the case where the periodicity of the Voronoi net with *R*_*f*_ ≥ 1.2 Å is 1D–3D, and the periodicity of the COP–COW net is 0D. There are no edges or nodes of the Voronoi net formed by atoms of only one molecule. For example, the structure of GANDUW, similar to GANDOQ, has a non-periodic intrinsic path through two molecules, but in this case the 12 *p*-fluorophenyl groups on the cage edges increase the distance between cages, which leads to a 1-periodic extrinsic pathway being created. The *p*-fluorophenyl groups of the surrounding molecules block the three other windows of the central cage such that the extrinsic pathway does not communicate with the intrinsic one.
4. Category 4, made up of 10 structures, corresponds to the case where the periodicity of the Voronoi net with *R*_*f*_ ≥ 1.2 Å is 1D–3D, the periodicity of the COP–COW net is 1D–3D, and there are two or more separate and inequivalent Voronoi nets. At least one of the two Voronoi nets passes through the intrinsic voids of the molecules in the structure (i.e. it contains edges and nodes of the Voronoi net formed by atoms of only one molecule), and its edges and nodes are formed by atoms of one molecule or pairs of molecules connected by a COW–COW edge. The 10 examples of this category are solvates of cyclic oligosaccharides (NUNRIX, OKUFOP, SIBJAO, SIBJES and TAHREZ), adamantane-like cages (NODWEK and OFOQEI), pillararene (MOXRAU), tubular trigonal prismatic cage (ABIMEG), and a co-crystal of tubular trigonal prism and adamantane-like cages (ABIQEK). Only ABIQEK and OFOQEI contain intrinsic and extrinsic pathways both of 3D periodicity, and NODWEK has 3D intrinsic and 1D extrinsic pathways, while all other examples have parallel 1D intrinsic channels.
5. Category 5 corresponds to all other cases, and it is the most frequent situation (443 structures). For example, two triptycene-based three-sided lantern cages in SATJAA and SATJEE [60] have large windows, but the packing of cages and the orientation of the windows are driven by the *π* · · · *π* stacking of the edges. As a result, more rigid conjugated edges in SATJAA enable denser packing with one closed and two open windows (leading to extrinsic 3D channels filled by solvent), while the addition of aliphatic groups into the edge of SATJEE prevents such stacking and opens all the three windows.

The 223 structures of molecules with inaccessible windows or intrinsic pores (diameters less than or equal to 2.4 Å) can only belong to one of two categories: 1 and 3. Molecules with accessible windows and intrinsic pores (diameters greater than or equal to 2.4 Å) are found in all of the five groups (810 structures). Thus, 184 structures in category 1 do not have migration pathways because of the presence of edge-to-face of corner-to-face molecule orientations. Nonetheless, most of the structures with accessible windows and pores enable migration of molecules through cages: 543 structures in categories 2, 4 and 5. In categories 2 and 4, the presence of intrinsic pathways is obligatory. However, in category 5, only 62 structures have such intrinsic pathways (defined by a 1D, 2D or 3D COP–COW net), meaning that in 381 structures, a migrating molecule would pass through a pathway including both intrinsic and extrinsic voidspace. This is the most common single case and highlights how both the cage structure and its crystal structure are necessary to create effective porosity.

### 3.5. Uncovering structural correlations

**Figure 9.**
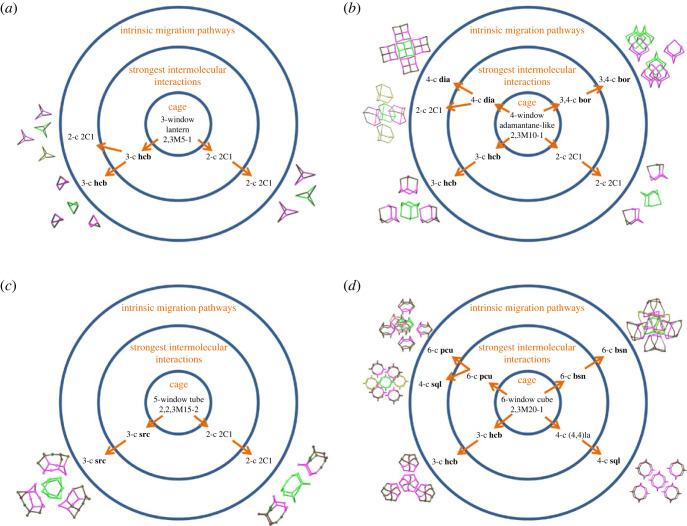
Relationship between cage skeleton topology and the resulting net of strongest intermolecular interactions and intrinsic migration pathways for (*a*) 2,3M5-1 3-window lantern cages, (*b*) 2,3M10-1 4-window adamantane cages, (*c*) 2,2,3M15-2 5-window tubular cages and (*d*) 2,3M20-1 6-window cubic cages.

From the data on the topological types of porous molecules, molecular clusters and molecular packings for quite a large set of structures, it is possible to extract correlations between the structures at the levels of each topological description. Table 1 shows the occurrence of migration pathway categories for common skeleton topologies found in our dataset. There is a non-random distribution of the periodic topology types over the migration pathway categories, which suggests that there is some relationship between the different levels of structure described in this work. These relationships are shown in figure 9 for the most frequent molecular skeletons.

**Table 1.** The occurrence of the most frequent molecular skeleton types in the migration pathway categories 1, 2, 3 and 5. The number of windows and the number of porous structures (with *R*_*w*_ and *R*_*p*_ > 1.2 Å) for each molecular skeleton are also shown.

skeleton topology	windows	structures with porous molecules	category	structures in the category	occurrence of skeleton type in category (%)
2,3M5-1	3	139	1	22	16
			3	16	12
			5	95	68
2M10-1	2	85	1	52	61
			2	19	22
2,3M10-1	4	116	1	16	14
			2	30	26
			5	63	54
2M8-1	2	28	3	12	43
			5	11	39
2M12-1	2	42	1	24	57
2M14-1	2	79	1	25	32
			3	13	17
			5	34	43
2,4M18-3	2	114	5	103	90
2M16-1	2	24	5	13	54
2,4M21-1	2	14	5	12	86
2,4M24-2	2	42	5	40	95

The most frequent types of skeleton topologies, such as 2,3M5-1 and 2,3M10-1, form migration pathways of different categories. The most prominent correlations between skeleton topologies and migration pathway categories can be found for eight types of skeletons; specifically, 2M10-1 and 2M12-1 form molecular packings with migration pathway category 1 (i.e. no migration pathway), and 2,4M24-2, 2,4M21-1, 2M16-1, 2,4M18-3, 2,3M5-1 and 2,3M10-1 form molecular packings with migration pathway category 5 (one migration pathway composed of intrinsic and extrinsic pores) with a probability greater than 50% in each case (table 1). For example, pillar[5]arene derivatives (with the skeleton topology 2M10-1) are found to have a high occurrence of structures with edge-to-face stacking of the porous molecules, which leads to a high occurrence (greater than 50%) of category 1 migration pathways (indicating an absence of accessible pathway) [61].

Only weak correlations exist in category 2 migration pathways (pathways through intrinsic pores only). The most frequent skeletons are 2,3M10-1, 2M10-1 and 2M12-1, but the probability of migration only through the windows for these skeletons does not exceed 30%. In this category, the pillar[5]arene molecules are packed in parallel columns with face-to-face orientation [62]. Three types of skeletons, 2,3M5-1, 2M14-1 and 2M8-1, form only extrinsic migration pathways (category 3) with probabilities of 12%, 17% and 43%, respectively. For example, the aliphatic side groups on the faces of the endo-functionalized molecular tube (skeleton type 2M8-1) in TIHCEV penetrate the windows of neighbouring molecules and also prohibit close packing of the molecules [63]. For the structures with separate migration pathways (category 4), almost all the skeletons are solitary with only 2M16-1 (γ-cyclodextrin) occurring in four structures. In general, the majority (53% in categories 2, 4 and 5) have migration pathways that penetrate the intrinsic voids of the molecules, but only 23 of 45 skeletons and their combinations have intrinsic migration pathways.

These trends in packing arrangement broadly agree with the physical interpretation that usually more spherical molecules tend to pack in spherical close-packing arrangements with coordination number 12 (face-centred cubic or hexagonal closest packing) or 14 (body-centred cubic packing), more elongated molecules (larger second momentum of inertia) tend to allow larger coordination numbers, and more regular and specific-shaped molecules (cube-like, tetrahedron-like, octahedron-like) show less diversity of packings and tend to have coordination numbers similar to the number of faces, as they largely pack in a face-to-face fashion.

Comparing the molecular packing topologies for migration pathway categories 1, 2, 3 and 5 shows that the most frequent topologies—**bcu-x**, **fcu**, **gpu-x** and 14T3—are found in all of the migration categories. When considering the local topology derived from only the strongest interactions, topological motifs of the well-known types 2C1, **hcb**, **dia** and (4,4)(0,2) are most frequent. This further supports the notion that the topology of molecular packing is not directly determined by the window and pore sizes of porous molecules, but rather a key factor in determining the periodicity of migration pathways is observing differences in the relative orientations of windows with respect to each other.

It is interesting to note that the COP–COW nets, which describe the connection of intrinsic pores (§3.4), in categories 2 (92 structures), 4 (8 structures) and 5 (62 structures) have the same topologies as those assigned to the molecular nets defined by only the strongest interactions (§3.2). Importantly, this suggests that there is a relationship between the packing topology of the strongest interactions and the topology of the intrinsic porosity found in our dataset of crystal structures. Furthermore, this relationship suggests that the strongest interactions (determined by a large solid angle or interaction surface area) favour a face-to-face (window-to-window) orientation of molecules. For example, figures 2*c*,*d* and 3*c* show the **dia** topology of the strongest interactions, which is the same as the topology of the COP–COW net. In total, 141 of 162 (87%) structures with intrinsic pathways (category 1) have the same COP–COW topology as the network of strongest interactions. These COP–COW topologies are mainly constructed by the cages of 17 skeleton topologies, from which the types 2,3M10-1 (adamantane-like cages), 2M10-1 (pillar[5]arene derivatives) and 2,3M5-1 (three-sided lanterns) are most frequent. In 9 and 12 of the remaining cases, the intrinsic migration pathways are subnets and supernets of the COP–COW net, respectively. In category 2, the most frequent topological types are 2C1 and **dia** (table 2). In category 4, only topologies 2C1, **dia** and **lon** are present, and in category 5 there are topologies 2C1, **bor** and **hcb**. These relationships between skeleton topology and topology of the porous network give some guide to enable designing of porous structures. The porous molecules with skeleton topologies 2,3M10-1, 2M10-1 and 2,3M5-1 would be intermediate goals of such a design. Other, less frequent, topologies of COP–COW nets (**cfc**, **pcu**, **lon**, (4,4)(0,2), **bsn** and **srs**) are also potentially interesting synthetic targets.

**Table 2.** The number of structures, *N*, of topological types of COP–COW nets in the categories of porous structures 2, 4 and 5.

category 2	*N*	category 4	*N*	category 5	*N*
1D 2-c 2C1	56	1D 2-c 2C1	7	1D 2-c 2C1	30
3D 4-c **dia**	26	3D 4-c dia	2	3D 3,4-c **bor**	12
3D 4-c **cfc**	2	3D 4-c lon	1	2D 3-c **hcb**	10
2D 3-c **hcb**	2			1D 3-c (4,4)(0,2)	2
3D 3,4-c **bor**	1			3D 6-c **pcu**	1
3D 6-c **bsn**	1			3D 4-c **dia**	1
3D 12-c **fcu**	1			2D 3-c **hcb** & 1D 2-c 2C1	1
3D 4,6-c **fsc**	1			3D 4-c **lon**	1
				2D 4-c **sql**	1
				3D 3-c **srs**	1
				3D 3-c **ths**	1

**Figure 10.**
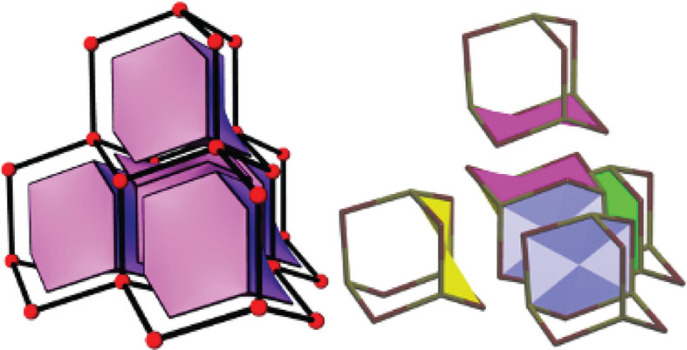
Tiling for the **dia** net with the tiles 6^4^ (left) and the packing of cages 2,3M10-1 (in FOXLAG) with the same motif of face fusion. The pairs of faces to be fused are highlighted in yellow, blue, magenta and green.

**Figure 11.**
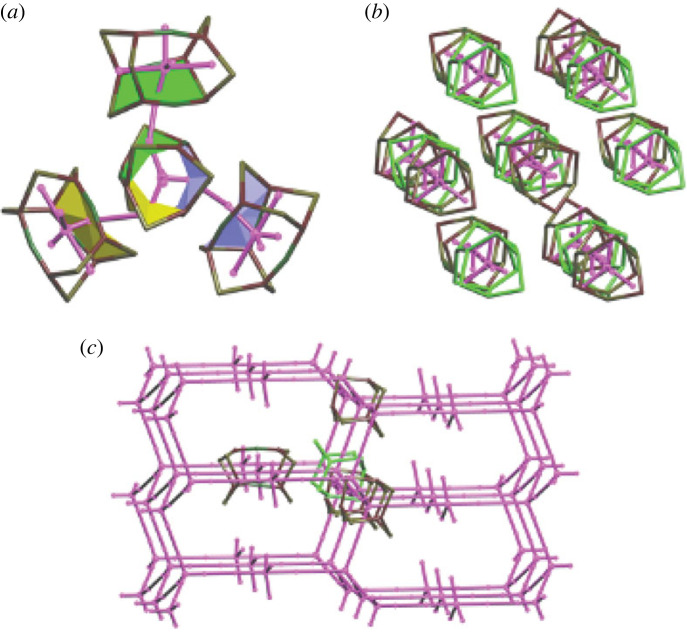
The skeletons and COP–COW nets (highlighted in magenta) illustrating the correlations between topologies of cages and intrinsic migration pathways: (*a*) 2,2,3M15-2—3D **srs** (ABIKUU), (*b*) 2,2,3M15-2—1D 2C1 (ABILAB) and (*c*) 2,3M10-1&2,2,3M15-2—3D **lon** (ABIQEK).

In this context, the face-to-face orientation of porous molecules is reminiscent of the tiling representation of porous networks [64] and assembling porous structures (e.g. zeolites) from tiles (cages fused by faces) [65]. For example, the hexagonal faces of the chair-shaped adamantane-like cages in the structure FOXLAG (figure 2) can be superimposed onto each other and their fusion would lead to the **dia** net, composed of the tiles of the same topology, 6^4^, as the 2,3M10-1 skeletons (figure 10). 2,2,3M15-2 with two 6-faces and three 8-faces (6^2^8^3^, which describes the number of nodes that make up a face of the skeleton) can be fused by three 8-faces into a porous framework in ABIKUU or alternatively by two 6-faces into infinite tubes in ABILAB. However, due to partial fusing (not all faces are shared), the remaining faces are located on the surface of extrinsic pores (figure 11). The COP–COW nets of ABIKUU and ABILAB have topologies of the 3D, 3-c net **srs** or 1D net 2C1, respectively. In the same way, adamantane-like cages can produce intrinsic pores of topologies 3D 3,4-c **bor** and 3-c **ths**, 2D 3-c **hcb**, and 1D 2-c 2C1. Moreover, it is even possible to co-crystallize molecules of two skeleton types, 2,3M10-1 and 2,2,3M15-2, to produce extended 2,4-c frameworks with 3D intrinsic pathways of underlying types **lon** (ABIQEK, ABIQIO) and **cfc** (ABILEF, ABILIJ) [66] and extrinsic channels with *R*_*f*_ = 3.73 Å, twice the size of the intrinsic channels (*R*_*f*_ = 1.70 Å). In these four structures (ABIQEK, ABIQIO, ABILEF and ABILIJ), the geometrically most compatible 6-ring chair-like faces come into contact. These examples are similar to those of reticular chemistry in MOFs, where the co-crystallization strategy enables tuning of the intrinsic pore topology and extrinsic pore size.

In our case, the intrinsic pore sizes are defined by the porous cages themselves and can only be tuned by changing the size of the porous molecules. For example, the structure UTEVOF, made up of enlarged adamantane-like cages, with *R*_*w*_ = 2.96 Å and *R*_*p*_ = 4.44 Å, has **dia** topology networks of strongest interactions and intrinsic pathways, which are similar to the networks seen in the structure OFOQOS, with *R*_*w*_ = 2.12 Å and *R*_*p*_ = 3.00 Å [10]. The corresponding sizes of intrinsic pathways also grow: *R*_*f*_ goes from 1.99 Å to 2.84 Å, and *R*_*i*_ goes from 2.91 Å to 4.37 Å in the structures UTEVOF and OFOQOS. An isoreticular relationship is found for UTEVOF and 29 other structures (entry 1 in table 3). Table 3 shows further examples of isoreticularity found in our dataset, which includes examples for networks of lower periodicity, QIXPIW and RAMPOL, and PIFVOS and PIFVAE, with molecular skeletons of type 2,3M5-1 and 2,3M10-1, respectively, and COP–COW nets with 2D **hcb** topology. Furthermore, the extension of tubular cages of topology 2,2,3M15-2 to produce isoreticular nanotubes with 2C1 intrinsic pathways is found in six structures (ABIMIK, ABIMOQ, ABIMUW, ABINAD, ABINEH and ABINIL). Unfortunately, such a strategy is not always successful. Interesting counterexamples to isoreticularity in porous molecular systems and their pore structures are the four structures with cages of type 2,3M20-1 (where the cage tiling is composed of six 4-ring faces, 4^6^), where the sizes of the cages differ significantly, leading to different COP–COW nets: PIFHIY (*R*_*w*_ = 3.83 Å, *R*_*f*_ = 2.41 Å) yields a 3D 4,6-c **fsc** net, PIFKIB (*R*_*w*_ = 4.66 Å, *R*_*f*_ = 4.42 Å) gives a 3D 6-c **bsn** net, PIFWAF (*R*_*w*_ = 2.71 Å, *R*_*f*_ = 1.92 Å) gives a 1D 3-c (4,4)(0,2), and PIFKEX (*R*_*w*_ = 3.79 Å), which has its windows open to extrinsic pores (*R*_*f*_ = 2.01 Å).

**Table 3.** The occurrences of isoreticular relationships between skeleton type and underlying net type, the number of structures, *N*, in our dataset that support the correlation, and the shape of the porous molecule.

correlation	molecule shape	*N*
2,3M10-1⟷**dia**	adamantane-like	29
2M10-1⟷2C1	cylinder-like	20
2,3M5-1⟷2C1	three-sided lantern	15
2,3M10-1⟷**bor**	adamantane-like	13
2M14-1⟷2C1	cylinder-like	11
2M12-1⟷2C1	cylinder-like	9
2M16-1⟷2C1	cylinder-like	8
2,3M10-1⟷**hcb**	adamantane-like	8
2,2,3M15-2⟷2C1	trigonal prism-like	6
2,3M10-1⟷2C1	adamantane-like	5
2M8-1⟷2C1	cylinder-like	4
2,3M5-1⟷**hcb**	three-sided lantern	3

## 4. Conclusions

We have used a series of material-agnostic definitions of topology to automatically analyse the topologies of molecular packing and pore networks in crystal structures of porous molecular materials. Our multi-scale topological analyses can be used to categorize porous molecular materials by their molecular skeleton topologies, local environments, crystalline packings and porous networks (or migration pathways). Using the underlying topological assignments to categorize porous molecules in our large dataset, we have explored the presence of structure–property relationships, and the extent to which isoreticularity holds, which may help guide future experimental endeavours. Unsurprisingly, the shape and external chemistry of porous molecular systems impacts their intermolecular interactions in subtle ways, leading to a delicate interplay between local and global structures, similar to that identified by a machine-learning investigation of molecular-based crystalline material porosity estimators [32]. By considering the topological relationship of the molecules, their windows and pores, we can distinguish three cases: polymorphism—a change in the topology of the migration pathways; breathing—a change in the migrating probe of extrinsic migration pathways; and responsive behaviour of molecules—a change in the migrating probe of intrinsic channels.

We found that while there was not a one-to-one relationship between the size and identity of the porous molecule and topology of the molecular packing, considering the packing of the *molecules* results in a variety of close-packed topologies such as **bcu-x**,**tcg-x** and **gpu-x**,**fcu**, very similar to that found for non-porous molecular crystals [26]. And consideration of the packing of the *pores* (i.e. the pore topologies) yields a prevalence of pore topologies (2C1, **dia**, **bor**, **hcb**, **lon** and **cfc**), featuring nets that are commonly found in MOFs (**dia**) and 2D-covalent organic frameworks (COFs) (**hcb**). The molecular packing of POCs is significantly determined by weak intermolecular interactions. Small changes to POCs, such as functionalizing linkers, may result in a different crystal structure, which may result in a different pore network. Therefore, any extractable ‘design rules’ are, necessarily, less strict observed correlations.

Nevertheless, we find that 3D migration pathways are possible with tubular, adamantane and cubic cages, which are likely to yield **srs**, **dia** and **pcu** migration pathways. 2D pathways, either hexagonal (**hcb**) or square planar (**sql**), are possible with all cage shapes, and 1D simple chains may result from lantern, adamantane and tubular cages, but are unlikely to form from cubic cage molecules. Overall, adamantane, cylinder, three-sided lantern and prism-shaped POCs are most likely to form crystals with intrinsic migration pathways.

Given a crystal structure of a POC, it is necessary to know two things in order to determine its likely use as an adsorbent: (i) the spatial arrangement of the intrinsic pores and (ii) whether molecular travel between those pores is possible. The spatial arrangement of pores is given by an analysis of molecular packing, undertaken here using ToposPro [31]. Using only the molecular packing is not sufficient, as the POCs may rotate or adopt a different conformer, such that molecules cannot pass from one pore to another. To resolve this, we recommend the net formed by linking the centres of each intrinsic pore via the centres of each cage window (the COP–COW net), the COP and COW are readily calculated using pyWindow [37] (code available at https://github.com/andrewtarzia/cage_collect/tree/master/pore_topologies) and then their net can be determined using ToposPro, in the same manner as one would determine the net of a MOF or COF. Comparing these two topological indicators can also be used to distinguish cases of polymorphism in POCs.

In the future, our database could be expanded to include other materials classes, such as metal–organic molecular systems, which, given the different intermolecular interactions present, may yield a different distribution of nets. Additionally, a more rigorous and automated search of crystal-structure databases could provide a more diverse set of molecules.

## Data Availability

The database of 1033 POC molecules is available at https://github.com/andrewtarzia/cage_collect/and a summary of the analysis is available at https://github.com/andrewtarzia/cage_datasets/tree/master/databases/POC_2019/summary. The pyWindow code for calculating the COP and COW is available at https://github.com/andrewtarzia/cage_collect/tree/master/pore_topologies. The Python code used to extract the structures from the CSD and run the analysis is freely available online at https://github.com/andrewtarzia/cage_collect/. The topological analysis of all database structures is included as a spreadsheet and all have been archived within the Zenodo repository: DOI:10.5281/zenodo.7362554 [67]. The data are provided in electronic supplementary material [68].
